# Size matters: micro-evolution in Polynesian rats highlights body size changes as initial stage in evolution

**DOI:** 10.7717/peerj.9076

**Published:** 2020-04-28

**Authors:** Alexandra A.E. van der Geer

**Affiliations:** Naturalis Biodiversity Center, Leiden, The Netherlands

**Keywords:** Cranial evolutionary allometry, Cranium, Geometric morphometrics, Insularity, Introduced species, Kiore, Muridae, Pacific rat, Rattus exulans, Ecological release

## Abstract

Microevolutionary patterns in populations of introduced rodent species have often been the focus of analytic studies for their potential relevance to understanding vertebrate evolution. The Polynesian rat (*Rattus exulans*) is an excellent proxy species because of its wide geographic and temporal distribution: its native and introduced combined range spans half the globe and it has been living for at least seven centuries wherever it was introduced. The objective of this study was to assess the effects of long-term isolation (insularity; up to 4,000 years) and geographic variables on skull shape variation using geometric morphometrics. A sample of 513 specimens from 103 islands and four mainland areas was analysed. This study, to my knowledge the first to extensively sample introduced rats, analysed 59 two-dimensional landmarks on the skull. Landmarks were obtained in three separate aspects (dorsal, lateral, ventral skull view). The coordinate data were then subjected to a multivariate ordination analysis (principal components analysis, or PCA), multivariate regressions, and a canonical variates analysis (CVA). Three measures of disparity were evaluated for each view. The results show that introduced Polynesian rats evolve skull shapes that conform to the general mammalian interspecific pattern of cranial evolutionary allometry (CREA), with proportionally longer snouts in larger specimens. In addition, larger skulls are more tubular in shape than the smaller skulls, which are more balloon-shaped with a rounder and wider braincase relative to those of large skulls. This difference is also observed between the sexes (sexual dimorphism), due to the slightly larger average male size. Large, tubular skulls with long snouts are typical for Polynesia and Remote Oceania, where no native mammals occur. The greater disparity of Polynesian rats on mammal species-poor islands (*’exulans*-only’ region) provides further insight into how diversity may affect diversification through ecological release from predators and competitors.

## Introduction

Microevolutionary patterns in populations of introduced rodent species have been the focus of analytic studies since the seminal work by R.J. Berry (e.g., Berry, 1963; Berry, 1964; Berry, 1969; Berry & Peters, 1975) as they have potential relevance to understanding vertebrate evolution (e.g., Patton, Yang & Myers, 1975; Yom-Tov, Yom-Tov & Moller, 1999; Pergams & Ashley, 2001; Cucchi et al., 2014; Renaud et al., 2018, and references therein). Microevolutionary processes ultimately underlie at least part of the macroevolutionary patterns (e.g., Oliver, 2013; Li et al., 2018); but see e.g., Gould et al., 1977; Gould, 2002; Sepkoski, 2012), who hold that macroevolution cannot always be explained by microevolution), and analysing them may shed light on the initial stages of evolutionary changes. Skull shape provides valuable information for investigating microevolutionary processes (Smith, 2011).

**Figure 1:**
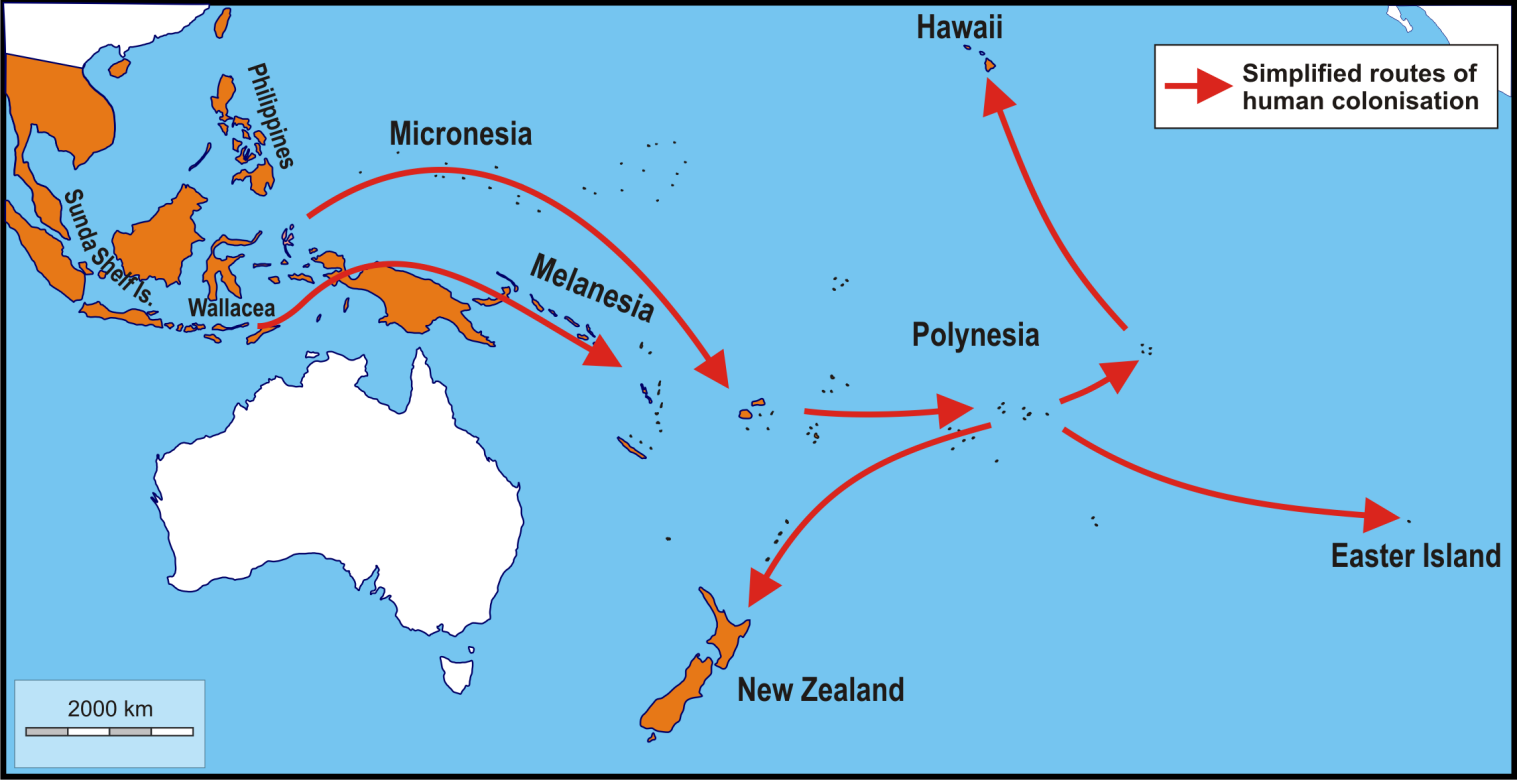
Schematic map showing simplified routes of human-aided dispersal of Polynesian rats, *Rattus exulans*. Populations on the Sunda Shelf Islands were isolated since the Last Glacial Maximum when sea level rises disconnected these areas from each other and the mainland. Populations in the Philippines and Wallacea were introduced about 4,000–3,500 BP. Subsequently, more remote areas were reached starting about 3,400–3,200 BP (Micronesia, Melanesia). The remotest areas of Polynesia (New Zealand, Hawaii and Easter Island), finally, were settled about 820–720 years ago (Roberts, 1991; Matisoo-Smith, Robins & Green, 2004; Wilmshurst et al., 2011; West et al., 2017). Not included are the very recent introductions to Taiwan and the southern Ryukyu Islands (arrival less than a century ago; Motokawa et al., 2001) and Adele Island, northern Australia (arrival c. 130 years ago; Boyle et al., 2004). Orange: native and introduced range. White: areas without Polynesian rats. Image credit: George Lyras. Map credit: https://d-maps.com/carte.php?num_car=3258&lang=en.

My aim here is to trace geographic variation in skull shape of populations of Polynesian rats (*Rattus exulans*), sampled from the greater part of its geographic distribution. The Polynesian rat is an excellent proxy species, because of its wide distribution: its native and introduced combined range spans half the globe and it has been living for at least seven centuries wherever it was introduced (Williams, 1973; Roberts, 1991; Corbet & Hill, 1992; Musser & Carleton, 2005; Wilmshurst et al., 2008; Wilmshurst et al., 2011; Fig. 1). Moreover, most of its combined range consists of islands, ranging in size from below 1 km^2^ for some atolls to over 785,000 km^2^ for New Guinea, in absolute latitude from 0° of the tropics and 50.5° for the Auckland Islands of New Zealand, and in maximal elevation from almost zero to over 5 km, again for New Guinea (Van der Geer, 2018). Polynesian rats are further an opportunistic species, able to exploit a variety of diets and habitats, ranging from rainforest to grasslands, under different climatic regimes, especially in the absence of other rodents (Dwyer, 1978; Rabor, 1977; Danielsen et al., 1994; Molur et al., 2005; Hingston, 2015). Islands further offer the possibility to test the effect of release from ecological competition and predation on skull shape, apart from skull size; in addition, microevolution in rodents appears to be faster on islands then on the mainland (Millien, 2006). Skull shape is quantified using geometric morphometrics. Application of geometric morphometrics in biology is well established (Zelditch, Swiderski & Sheets, 2012), and is a powerful tool for intraspecific discrimination in rats and mice, as well as on the mainland as in restricted areas such as islands (e.g., Valenzuela et al., 2009; Claude, 2013; Cucchi et al., 2006; Cucchi et al., 2013); Hulme-Beaman et al. 2018; Hulme-Beaman et al. 2019. Claude (2013) showed that Procrustes coordinates (and teeth size) are better in discriminating between Polynesian rats and the Asian house rat (*Rattus tanezumi*) of Thailand than other morphometric data.

Genomic data point to the island of Flores, one of the Lesser Sunda Islands, Indonesia, as the place of origin of the Polynesian rats (Thomson et al., 2014). This was already suggested by Schwarz (1960), as opposed to the mainland of Southeast Asia (Tate, 1935), based on the presence on Flores of white-bellied individuals. Schwarz & Schwarz (1967) considered the white-bellied form the wild ancestral form of all commensal types, and the loss of a white belly as an adaptation to the darker indoor environment. However, there are no significant morphological differences between populations from Flores and the rest of the Lesser Sunda Islands (Musser, 1981). Furthermore, despite the rich murid fossil record going back to the Middle Pleistocene, no fossil or subfossil Polynesian rat has yet been reported from Flores. The exact place of origin may have important implications for tracing human migrations through insular Southeast Asia, but has much less relevance to the classification of skull shape. Complicating matters further, the dispersal of Polynesian rats was primarily facilitated by the ancestral Polynesians, who started out from Taiwan, and the rats they took aboard their vessels likely were already commensal and need not have come directly from Flores. The main objective here is not to trace the potential routes of dispersal, nor to trace the place of evolutionary origin of this species, but to explore shape variation in response to the colonisation of the various islands.

**Figure 2:**
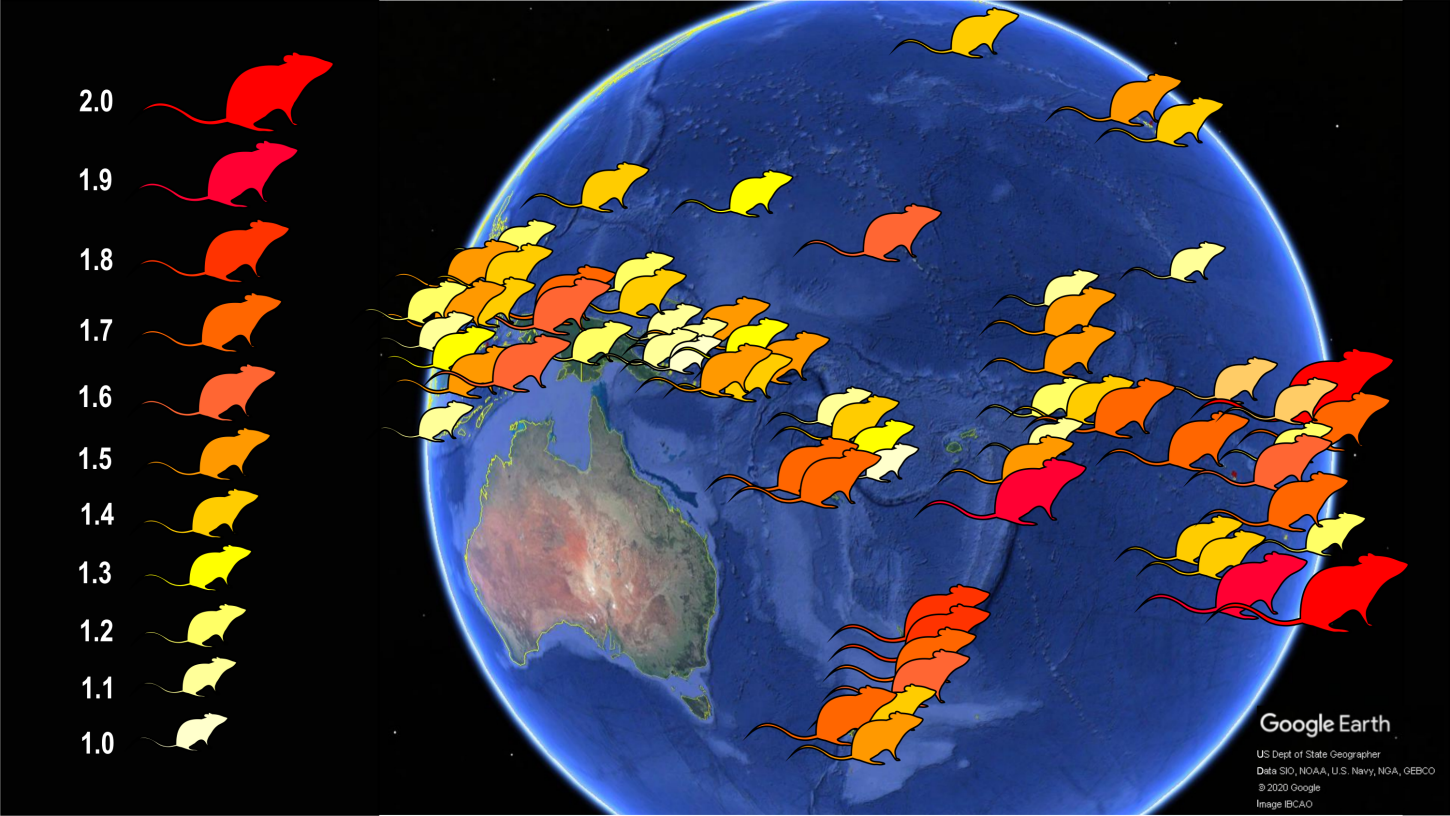
Schematic map showing the body size increase of Polynesian rats on Pacific islands. Populations of these commensal rats on islands, in contrast to the mainland, evolved larger body size, with the largest rats in temperate New Zealand and tropical atolls of the Pacific. The number to the left of the rat symbols indicate body size relative to the average body size of mainland rats. Data from Van der Geer (2018). Image credit: George Lyras. Map credit: Google Earth, US Dept of State Geographer, Data SIO, NOAA, U.S. Navy, NGA, GEBCO, c 2020 Google, image IBCAO. Silhouette credit: Phylopic (2020).

To this goal, I explored skull shape variation in correlation with skull size (a proxy for body mass), latitude (a proxy for climate), island area, elevation (a proxy for habitat diversity), geographic grouping (a proxy for time in isolation), and number of native ecologically relevant competitors and predators. Earlier studies on island mammals have shown that these variables have a predictive effect on body size evolution in native mammals (e.g., Lomolino, 1985; Lomolino, 2005; Lomolino et al., 2012; Lomolino et al., 2013), as well as in introduced mammals (e.g., Van der Geer, Lomolino & Lyras, 2018; Van der Geer, 2018), including the Polynesian rat (Fig. 2). Body size, and indirectly skull size, is on average larger in populations experiencing reduced competitor and predator stress through relaxed selection (Whittaker & Fernandez-Palacios, 2007; Lomolino, Riddle & Whittaker, 2017), on small or low islands (Lomolino et al., 2012; Van der Geer, 2018) and in those with greater residence times, or in the lower latitudes (Lomolino et al., 2013; Van der Geer, Lomolino & Lyras, 2018). The main objective here is to test if these variables have a predictable effect on shape as well, in addition to size, and if so, in what direction. Another objective is to explore if and to what extent the trend of cranial evolutionary allometry (CREA) can be observed in insular Polynesian rats in relation to their increased body size. In mammals, the region of the muzzle is proportionally longer than that of the braincase in larger species (e.g., Gould, 1966; Schluter, 1996; Radinsky, 1985; Cardini & Polly, 2013; Tamagnini, Meloro & Cardini, 2017; Cardini, 2019). This allometric pattern is widespread among mammals (eutherian as well as metatherian), but has to my knowledge not been tested in introduced mammal species.

## Materials & Methods

Cleaned skulls of wild-caught adult Polynesian or Pacific rats, *Rattus exulans*, were used (for specimen numbers and locality data, see Data S1). Total number of specimens was 513, of which 28 from the mainland (Malaya, Laos, Thailand, Vietnam),and 485 from in total 103 islands. Where specimen numbers allowed, only adult specimens with advanced dental wear (stages IV or V; Karnoukhova, 1971; King, 2006) were analysed, as rats continue to grow slightly after they reach maturity (Roach et al., 2003).

**Figure 3:**
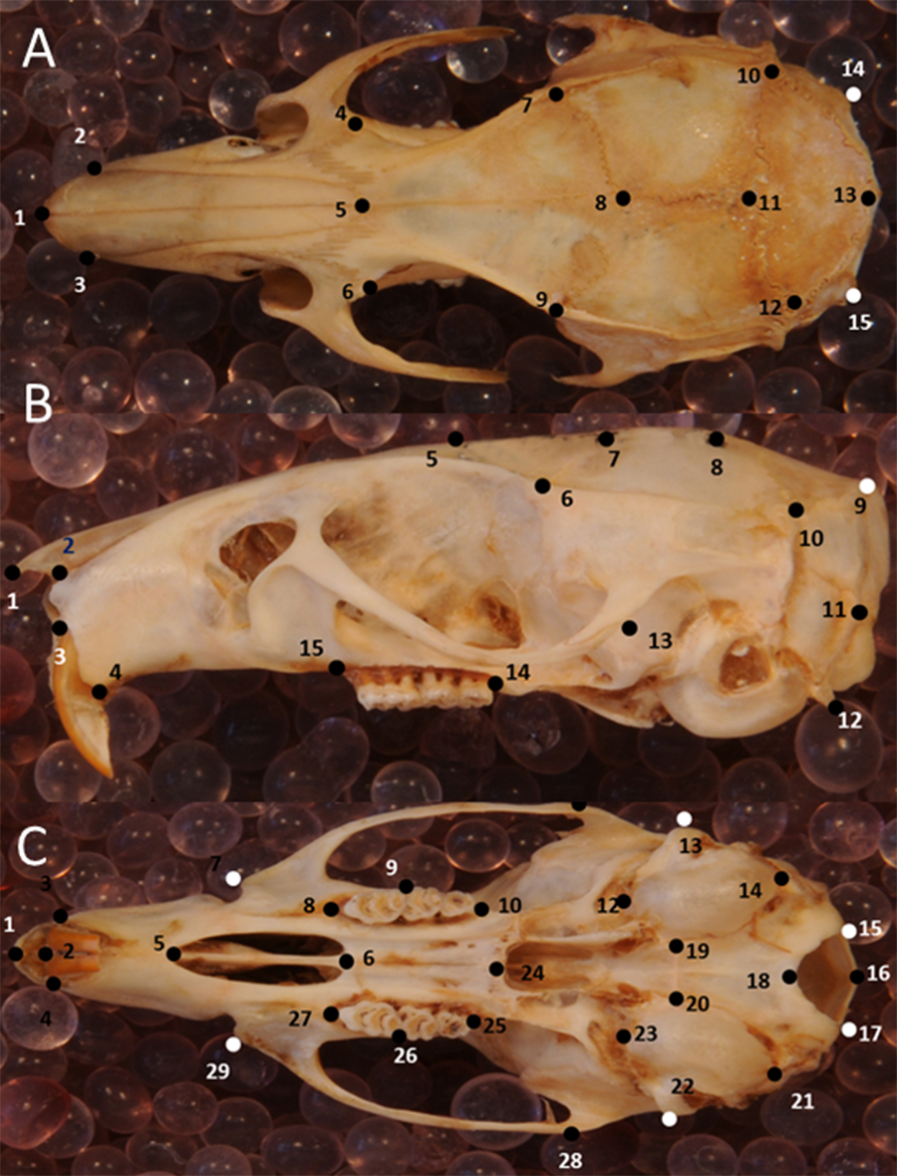
Position of the landmarks used in this study on photographs of Polynesian rat skulls. Location of landmarks (numbered black or white points, colour depending on the background) for geometric morphometric analysis on cleaned, adult skulls of *Rattus exulans*. (A) Dorsal view. (B) Lateral view. (C) Ventral view. Definitions of the individual landmarks are given in Table S1.

Skull shape was quantified with geometric morphometrics using 2D landmarks. A key advantage of this method is that shape variation can be visualized directly (Rohlf & Marcus, 1993). The error resulting from the 3D to 2D approximation in geometric morphometric studies proved to be negligible when tested for a rodent skull (hoary marmots, *Marmota caligata*; (Cardini, 2014). Three skull views (dorsal, lateral, ventral) were explored, with 15, 15 and 29 landmarks, respectively (Figs. 3A–3C, Table S1). To this end, photographs were taken of the dorsal, left lateral and ventral perspective of skulls separately for all specimens. Occasionally, photographs of the right lateral side of the specimen were taken and then mirrored, when the left side was too damaged or incomplete. A setup of a digital camera (Nikon D300S) fitted with an EF 100 mm f/2.8 macro lens and mounted on a photographic copy stand was used in combination with a remote control to avoid camera shake. Light sources were placed at each corner. I used a standard container filled with silica balls to hold the specimens in position and a spirit level to ensure that they lay in a flat plane relative to the lens. A mm ruler fixed onto the container was oriented along the long axis of the specimen, and skulls were aligned so that the sagittal suture (dorsal view), the zygomatic arch (lateral view) and midpalatal suture (ventral view) were aligned with the ruler. (lateral view). After all pictures had been collected, landmarks were digitized in tpsDig2 (Rohlf, 2006), version 2.32, for all specimens. Landmarks followed Corti & Rohlf (2001); house mice), Kawakami & Yamamura (2008); house mice), Maestri et al. (2016); rodents); Singh et al. (2017); brown rats, *Rattus norvegicus*).

Shape is defined as the resulting geometry after the size, location and orientation has been removed from the landmark data (Kendall, 1984), as well as any departure from perfect bilateral symmetry (Mardia, Bookstein & Moreton, 2000), because asymmetry may result from developmental disturbances due to environmental factors (Van Valen, 1962). To achieve this, a full generalized Procrustes analysis (Dryden & Mardia, 2008) with object symmetry (Klingenberg, Barluenga & Meyer, 2002) was performed on the collected coordinates using the software MorphoJ (Klingenberg, 2011). Size was approximated using centroid size of the specimens, which is the square root of the sum of squared Euclidean distances from each landmark to their own centroid (Dryden & Mardia, 2008). Principal components are calculated in order of the amount of variation they cover, where PC1 captures the most variation, PC2 the second most and so on.

Canonical variates analysis (CVA) was used to calculate the Procrustes distances between grouped samples. The major purpose of canonical analysis is to maximize differences between groups by producing weighted variables, referred to as canonical variates (Pietrusewsky, 2008). Generally, the first few canonical variates describe most of the variation present, analogue to principal component analysis. I here use the function Canonical Variate Analysis in MorphoJ to visually represent the differences among pre-assigned groups (see below and Data S1).

For the PCA and CVA, eigenvalues of each component or variate that explained at least 10% of the total skull shape variation within each view were considered significant for further interpretation. Shape changes for each principal component and canonical variate were visualized using the warped wireframe function in MorphoJ.

Multivariate regressions were performed in the software Past 3.16 (Hammer, Harper & Ryan, 2001) on each dataset to examine the effect of skull size, using centroid size, latitude, maximal elevation and island area on the skull shape variation as shown in the Procrustes coordinates. A permutation test with 10,000 randomization rounds was performed on each regression to test for independence, and the significance of correlations was tested with a Shapiro–Wilk test (Shapiro & Wilk’s, 1965). Significance level for all tests *p* = 0.05.

The geographical grouping (see Fig. S1) is an adaptation after Hingston (2015), which is based on a combination of genomic data of Polynesian rats (mainly mitochondrial haplotypes) and biogeography, reflecting the composition of the native flora and fauna as well as the average time frame of introduction, with the addition of New Zealand as a separate region. The ten groups are the following, in alphabetical order, Inter Oceania, Island Papua New Guinea, Mainland (of Southeast Asia), Moluccas, Micronesia, New Guinea, New Zealand, Philippines-Borneo-Sulawesi, Remote Oceania and the Southern Malay Archipelago (Fig. S1).

Only ecologically relevant native mammalian predators and competitors are taken into account, based on published fauna lists (Van der Geer, Lomolino & Lyras, 2017; Van der Geer, Lomolino & Lyras, 2018). The number of predators was binned as zero, 1–2, 3 or more predators; the number of competitors was binned as zero, 1–4, 5 or more competitors (see Data S1). Geographical data (surface area (log_10_ km^2^), maximum elevation (m) and latitude) were taken from (Van der Geer, Lomolino & Lyras, 2018); for islands not included in the latter, data were from the Islands Website of the United Nations Environment Programme (http://islands.unep.ch/). Phylogenetic information (mtDNA haplotypes; Data S1) is after Matisoo-Smith, Robins & Green (2004), Thomson et al. (2014), and (West et al., 2017).

Institutional abbreviations: AMNH = American Museum of Natural History (New York, USA), USNM = Smithsonian Institute, Natural History Museum (Washington, USA), and NMNZ = Te Papa Tongarewa Museum of New Zealand (Wellington, New Zealand).

## Results

For raw data, see Data S2 (dorsal skull aspect), Data S3 (lateral aspect) and Data S4 (ventral aspect). Note: all tests are significant at the 0.01–0.0001 level based on 1000 permutations unless specified otherwise.

### Dorsal view

**Figure 4:**
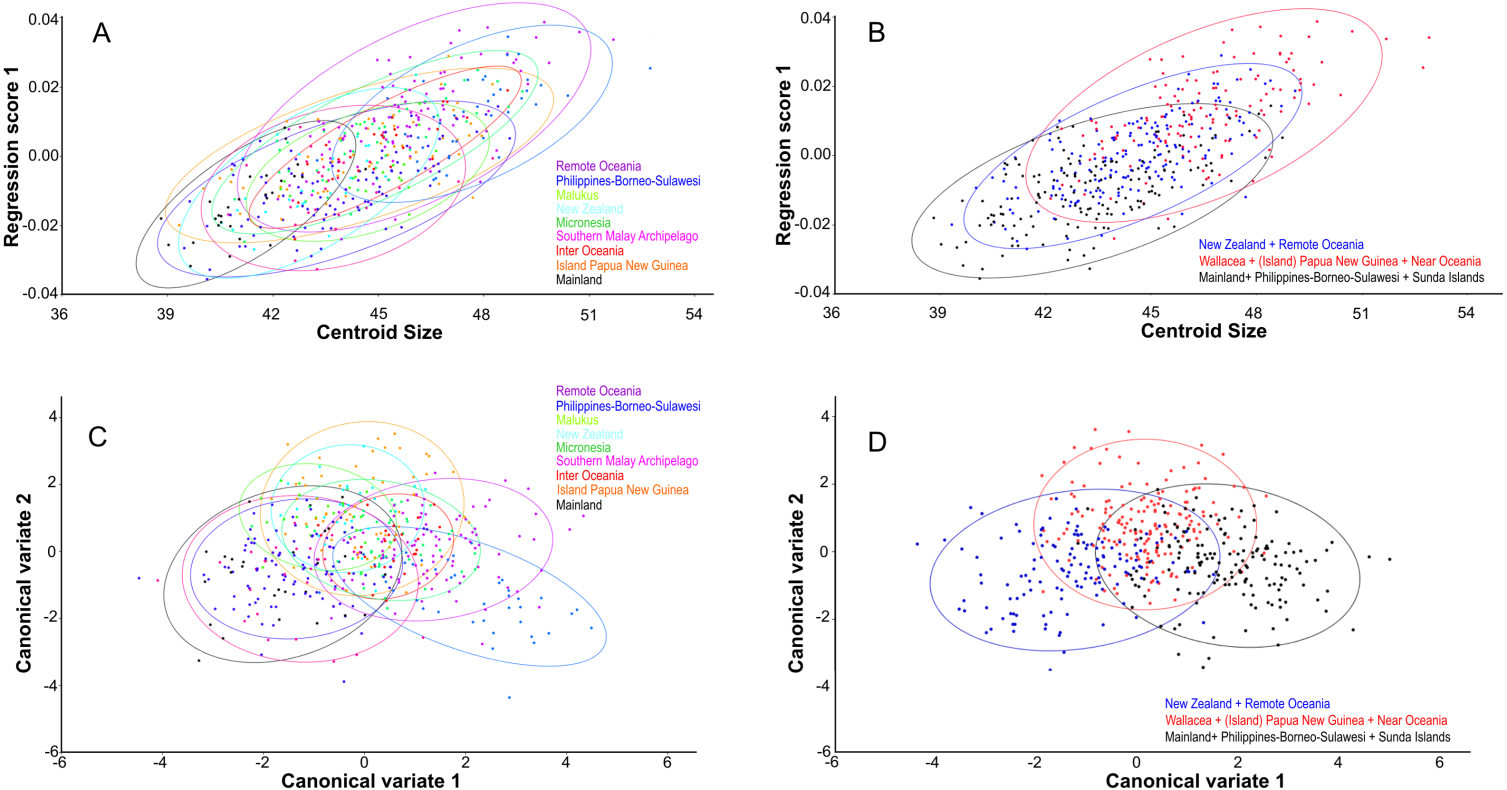
Geographic shape variation in skulls of Polynesian rats. Dorsal aspect. (A) Overall shape, taking all PC’s into account, regressed over centroid size, following the ten geographical groups. (B) As (A), but with the groupings simplified and reduced to three major groupings. (C) Shape variation as shown by canonical variate analysis shows a similar broad overlap between individual groupings. (D) as (C), but with the groupings simplified and reduced to three major groupings.

**Figure 5:**
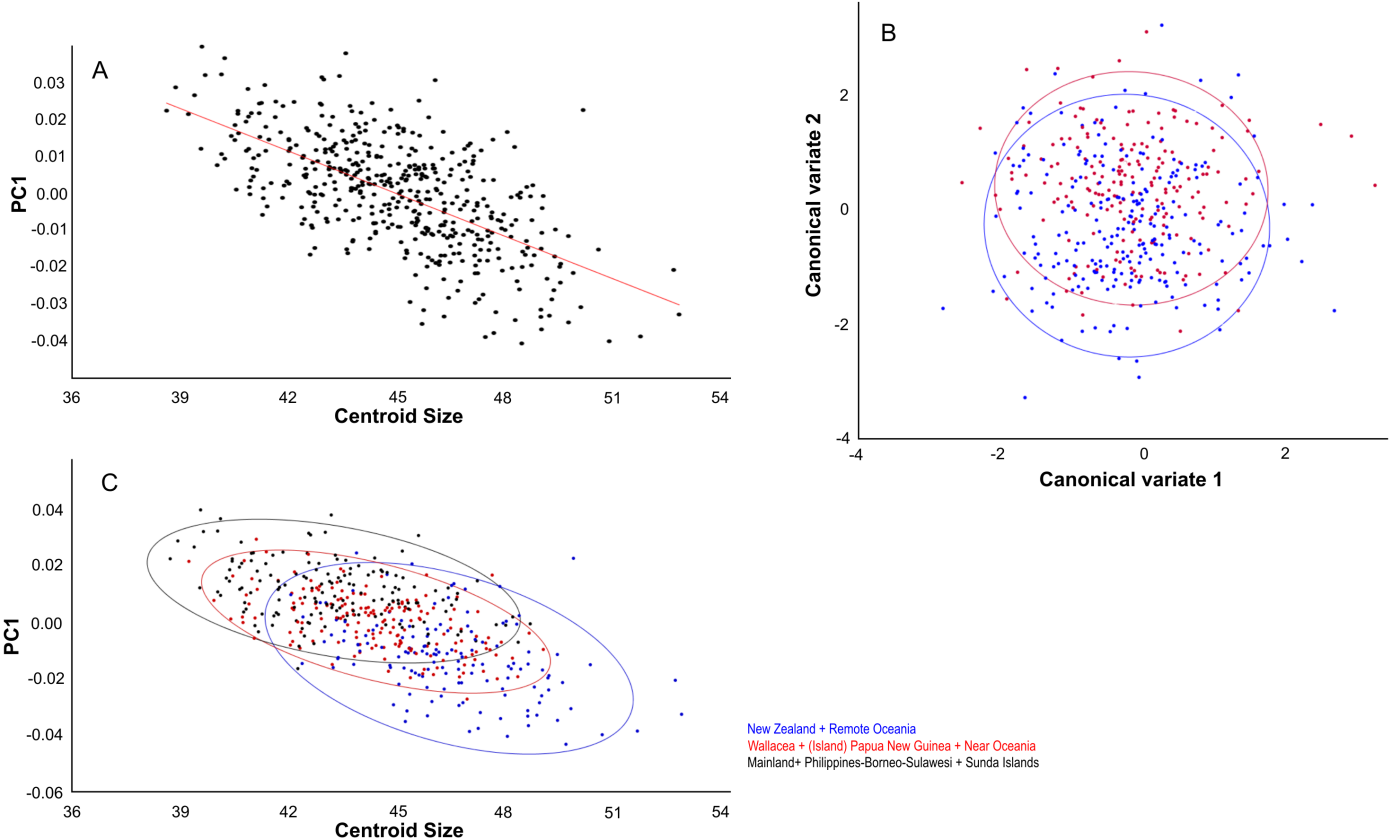
Effects of gender and size on skull shape. Dorsal aspect. (A) Linear regressions of PC1 shape and centroid size. (B) Canonical variate analyses of overall shape separates the genders. (C) The largest specimens are from New Zealand and Remote Oceania and have overall lower PC1 scores. Colour codes: (B) Blue = males. Red = females.

Centroid size predicts explains 14% of the total variation in dorsal shape space.. The shape variation differs among the ten groupings (Fig. 4A), but can be simplified and reduced into three major geographical groupings, which are as follows: (1) Mainland, Philippines-Borneo-Sulawesi, and the Southern Malay Archipelago, (2) the Moluccas (Malukus), (Island) Papua New Guinea, Micronesia, and Inter Oceania, and (3) New Zealand and Remote Oceania (Fig. 4B). Canonical variate analysis also shows this geographical grouping (Figs. 4C, 4D; Figs. S2A, S2B) with skulls from New Zealand and Remote Oceania having relatively narrow and longer, ‘tubular’ skulls, as mainly described by CV1 (54% of the variance; Fig. S2A). This major grouping roughly coincides with the distribution of the haplotypes (1, 2, 3A and 3B combined). The main difference between the sexes is best explained by CV2 (21% of the variance), with females having rounder skulls with shorter muzzles and with the widest point at the level of the frontals (as in PC3; see below) (Fig. 5A; Figs. S2C, Figs. S2D). The effect of competition and predation is expressed as higher CV1 scores without predators and competitors, whereas the number of predators/competitors, when present, has not much effect (Figs. S3A– S3C).

**Figure 6:**
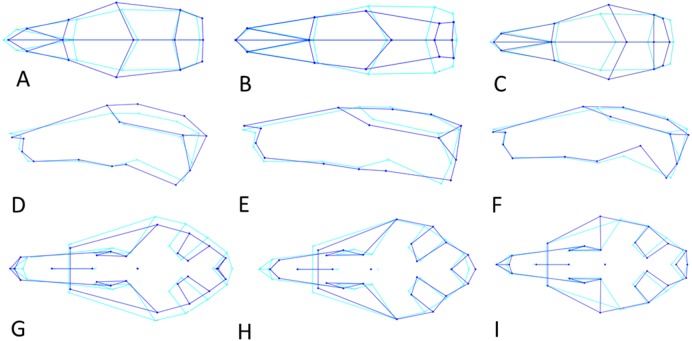
Shape changes along the three Principal Component vectors. Shape changes in light (average shape) and dark blue (maximum change in positive direction). (A–C) Dorsal view. (D–F) Lateral view. (G-I) Ventral view. (A, D, G) First principal component (PC1). (B, E, H) Second principal component (PC2). (C, F, H) Third principal component (PC3).

The distribution of specimens in shape space is largely influenced by PC1. PC1 explains 29% of the variance and mainly describes variation in muzzle length and width of the anterior part of the neurocranium (Fig. 6A). Skulls with a high PC1 score have shorter muzzles and a broader skull. Centroid size predicts 40% of the PC1 shape variation, and is negatively correlated with PC1 (Pearson *r* =  − 0.63; *p* < 0.001) (Fig. 5B). Males have marginally lower PC1 scores: average PC1 is -0.00198 for males (*n* = 231), and 0.00232 (*n* = 197) for females, with a standard error of 0.001 for both. This is likely a direct result of their slightly larger body masses: average centroid size is 45.21 for males (*n* = 231, standard deviation = 2.31) and 43.71 for females (*n* = 197, standard deviation = 2.09). The difference between males and females is also reflected in the CV1 (79% of total variance) of the canonical variate analysis as longer muzzles and narrower skulls (Fig. 5C; Fig. S2C). The same applies to specimens from Remote Oceania and New Zealand: they are separated from those from the mainland, Southern Malay Archipelago, Philippines, Borneo and Sulawesi on the one side and the islands of the rest of Indonesia, New Guinea, Micronesia and Near Oceania on the other side, based on their lower PC1 scores in combination with a higher centroid size (Fig. 5D).

**Figure 7:**
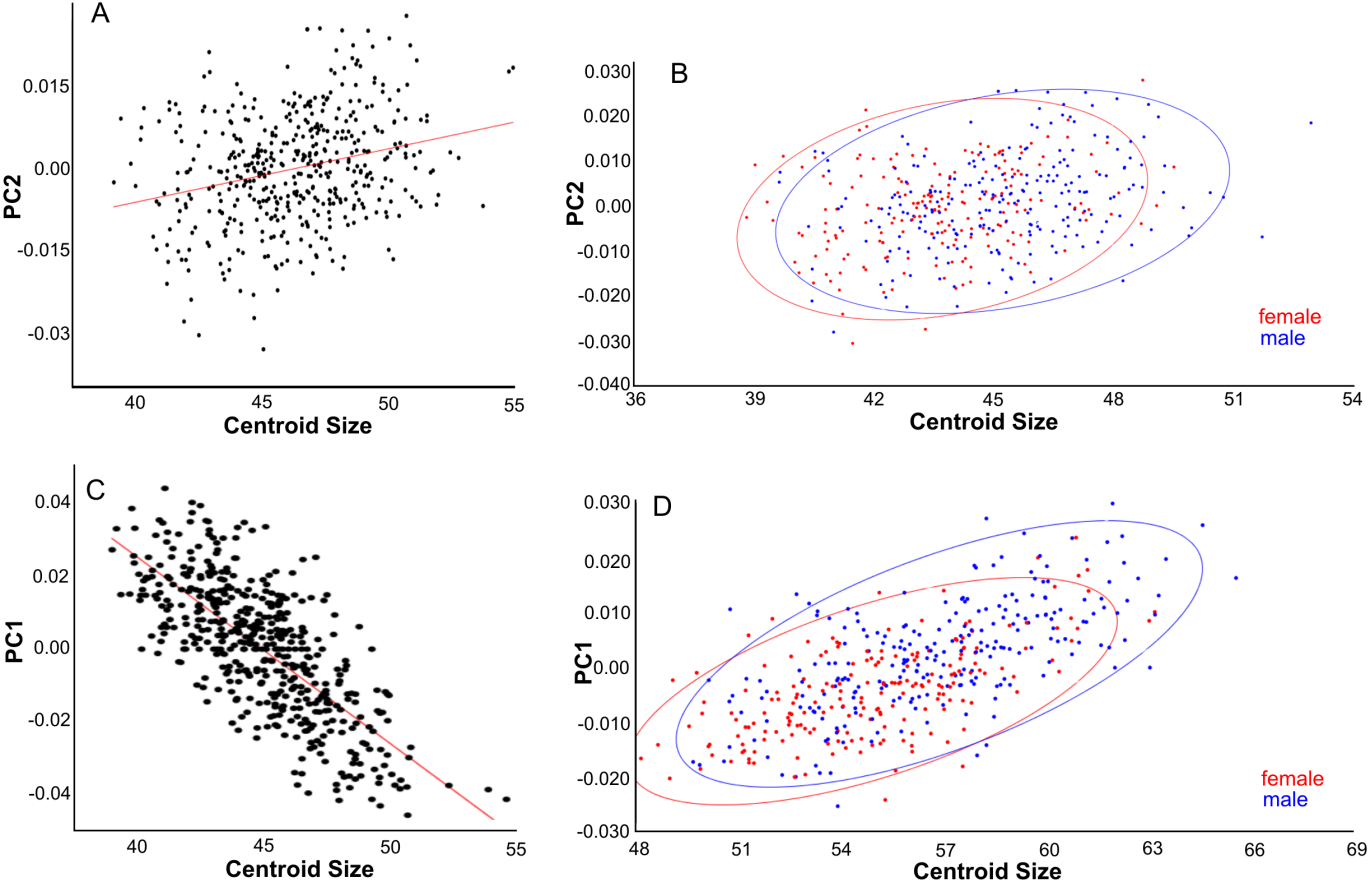
Effects of size and gender on skull shape. (A, B) Dorsal aspect. (C) Lateral aspect. (D) Ventral aspect. (A) Linear regressions of PC2 shape and centroid size. (B) PC2 scores are slightly higher for males than females due to their larger size. (C) Linear regression of PC1 shape and centroid size. (D) PC1 scores are higher for males than for females, partly due to their larger size.

PC2 explains 13% of variance and describes variation in the width of the posterior neurocranium (temporal region) and the relative size (length and width combined) of the occiput (Fig. 6B). Skulls with a high PC2 score have narrower skulls with longer temporal bones but shorter occipital bones. PC2 is positively correlated to centroid size (Pearson *r* = 0.27, *p* < 0.001), where centroid size predicts 7% of the variation in PC2 (Fig. 7A). Sexual dimorphism thus predicts marginally PC2: females have, on average, a lower PC2 value compared with males (’balloon-shaped’; PC2 score is −0.0012 and 0.0010, respectively; standard error 0.001 in both cases) (Fig. 7B).

PC3 (12% of variance) describes the variation in the width of the neurocranium at the level of the frontotemporal sutures (Fig. 6C). Skulls with a high PC3 score have a wider skull at the frontals, resulting in a rounder skull (’balloon shape’). Centroid size is negatively correlated with PC3 (*p* < 0.001; Pearson *r* =  − 0.24), and predicts 6% of the shape variation. Female skulls tend to be broader at the frontals, due to their smaller size (average PC3 score is −0.0011 for males and 0.0013 for females; standard error 0.001). Latitude predicts 4% of the variation, with rounder skulls in lower latitudes, counter to predictions following Allen’s rule, but in agreement with body mass distribution patterns.

### Lateral view

**Figure 8:**
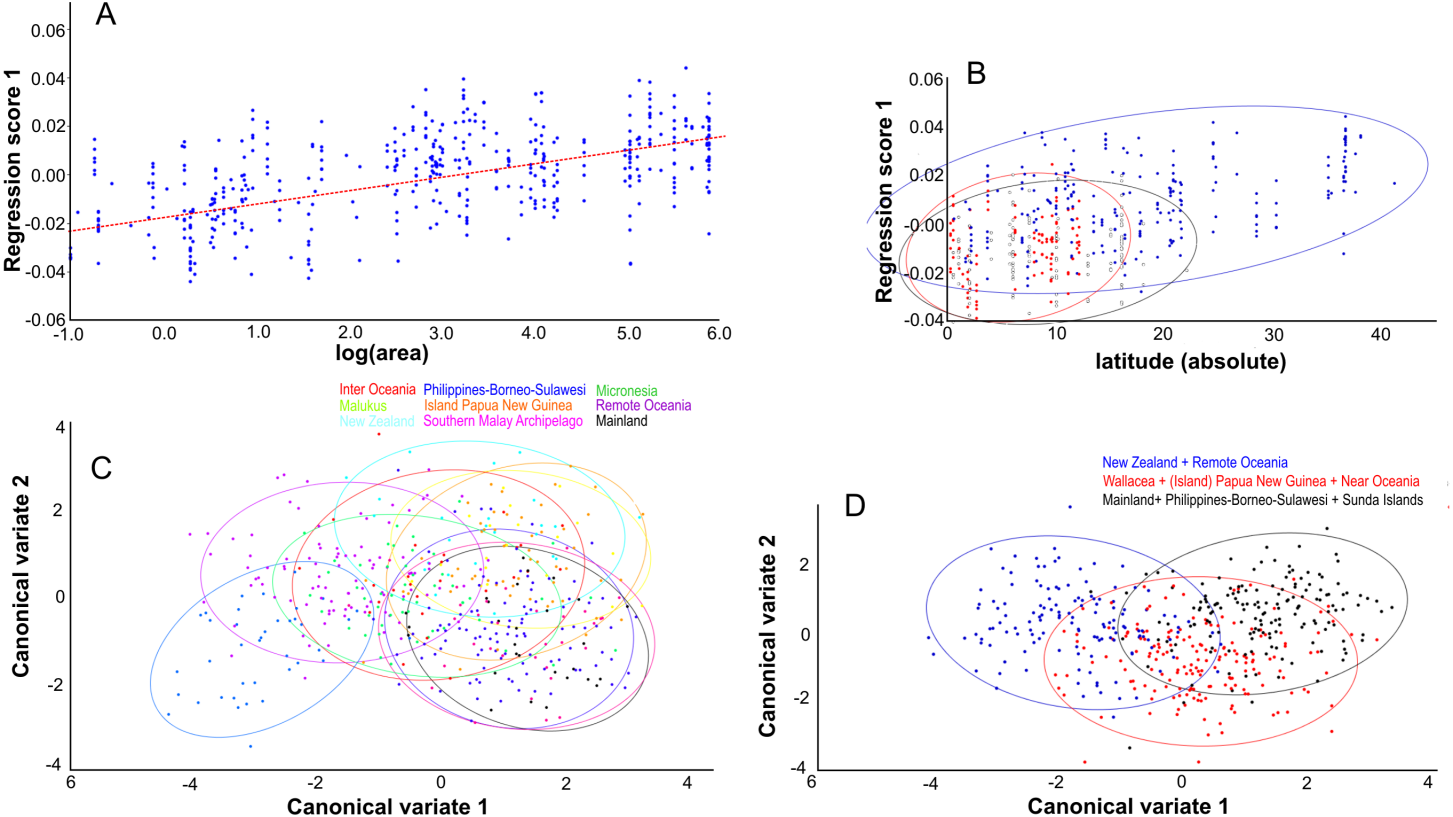
The effect of island area, latitude and geography on shape. Lateral aspect of the skull. (A) Area is positively correlated with overall skull shape (*r*2 = 0.076, *p* < 0.0001), here explained as a side-effect of competition and predation pressure (see Discussion). (B) Latitude is positively correlated with overall skull shape (*r*2 = 0.27, *p* = 0.0001), mainly due to the effect of competition, where islands in the higher latitudes lack native competitors (*exulans*-only part of the distribution of the Polynesian rat). (C) Canonical variate analyses separates the ten geographical groupings with much overlap. (D) As (C), but with the groupings simplified and reduced to three major groupings.

Centroid size predicts 15% of the total variation in lateral shape (*p* < 0.0001). This is in agreement with the correlation between the total shape variation, taken all PC’s into account, and island area (*r*2 = 0.076, *p* < .0001; Fig. 8A), and absolute latitude (*r*2 = 0.2700, *p* = 0.0001), the effect of which appears to be driven by ecological competition (Fig. 8B). Canonical variate analysis separates the regions with much overlap, similar to the results from the dorsal view (Figs. 8C, 8D; Figs. S2E, S2F). The most striking shape difference is the flatter, tubular skulls from Polynesia compared to the higher and less tubular skulls of the mainland, Sunda, Melanesia and Wallacea. Canonical variate analysis further illustrates the difference between the genders, with males having a flat, low skull as opposed to the high, more rounded skulls of the females, as explained by PC1 (see below) (Figs. S2G, S2H).

PC1 explains 27% of the variance and mainly describes variation in muzzle length respective to skull height and zygomatic length (Fig. 6D). Skulls with a high PC1 score have a shorter muzzle, are higher and have a shorter zygomatic process. Centroid size predicts 54% of this variation, and is negatively correlated with PC1 (Pearson *r* =  − 0.73, *p* ≪ 0.001) (Fig. 7C). Males have, on average, slightly lower PC1 scores (lower skulls, tubular shape), in accordance with their larger body masses (average PC1 is −0.0021 for males and 0.0054 for females; standard deviation = 0.017 and 0.018 resp.). The distribution of specimens from the various regions in PC1 space shape roughly follows the body mass distributions, although with considerable overlap, where specimens from Remote Oceania and New Zealand tend to have the lowest PC values, and those from the Mainland, Southern Malay Archipelago and Philippines-Borneo-Sulawesi the highest PC values (Fig. S4A). The space shape of the various subspecies follows these groupings reasonably well, with *exulans* of New Zealand and Remote Oceania having the lowest PC1 values and *concolor* (mainland), *ephippium* (Sumatra, Java, Bali, Borneo) and *raveni* (Sulawesi) having the highest values (Fig. S4B).

PC2 explains 12% of the variance and mainly describes the variation in proportional muzzle length (Fig. 6E). Skulls with a high PC2 score have longer muzzles. Centroid size does not predict PC2 variation (0.2%, Pearson *r* = 0.0459, *p* = 0.3352). PC2 mainly explains sexual dimorphism, with males having higher scores (average PC2 is 0.0019 for males and −0.0022 for females; standard deviation = 0.0124 and 0.0115, respectively): males have longer muzzles than females at the same size (Shapiro–Wilkinson test indicates a normal distribution, *p* = 0.57; unpaired *t*-test, *p* = 0.00079).

PC3 explains 10% of the variance and mainly describes the variation in the length of the zygomatic process of the temporal bone relative to muzzle length (Fig. 6F). Skulls with a high PC3 score have a shorter snout and a shorter zygomatic process Centroid size is not correlated to the variation in PC3 (*p* = 0.63, *r*2 = 0.0005). Males and females cannot be separated in PC3 shape space.

### Ventral view

Centroid size predicts 11% of the total variation in ventral shape (*p* < 0.0001). Latitude is positively correlated to the total variation, but explains only 4%. The other variables had no predictive value.

PC1 explains 20% of the total variance and describes the width of the neurocranium and the length of the muzzle (Fig. 6G). High PC1 scores represent skulls with a longer muzzle and a narrower braincase (’tubular shape’). They also have a proportionally shorter first molar and shorter total tooth row. The projection area of the foramen magnum on the ventral profile scales positively with skull width. PC1 is positively and strongly correlated to centroid size (Pearson *r* = 0.70), and centroid size predicts 49% of the variation (*p* < 0.0001). Males and females are marginally separated in PC1 shape space, in accordance with their different average centroid size (Fig. 7D). Latitude predicts only 6.5% of the variation in PC1 (Pearson *r* = 0.25) (Fig. S5A).

PC2 explains 12% of the total variance and describes the width of the zygomatic arches, and the orientation and shape of the auditory bullae (Fig. 6H). Skulls with a high PC2 value have slightly more closed zygomatic arches, and more elliptical, slightly larger and more obliquely placed auditory bullae. PC2 is positively but weakly correlated with centroid size and latitude (Pearson *r* = 0.19 and *r* = 0.26, respectively): centroid size and latitude predict 3.5% and 7% of the variation in PC2 (*p* < 0.001) (Figs. S5B, S5C). Polynesian rats of the higher latitudes, mainly New Zealand and its islands, are larger and have a higher percentage of skulls with narrower zygomatic arches, and elongated, somewhat larger bullae.

PC3 explains 11% of the total variance and describes the relative length of the basioccipital and position of the palate (Fig. 6I). Skulls with a high PC3 score have a shorter basioccipital bones and a more posteriorly placed palate. Centroid size (Fig. S5D) and latitude are not correlated with PC3 (*p* = 0.76 and *p* = 0.20 respectively). PC3 does not separate the genders.

## Discussion

Geometric morphometric analyses applied in this study showed that the effect of skull size on skull shape as reflected in PC1 of the three different views is strong in insular Polynesian rats. This is in agreement with earlier findings for mammals, according to which the region of the face is proportionally longer than that of the braincase in larger species. (e.g., Gould, 1966; Schluter, 1996; Radinsky, 1985; Cardini & Polly, 2013; Tamagnini, Meloro & Cardini, 2017; Cardini, 2019). Overall, with increasing body mass, skulls get longer, narrower and lower, resulting in a tubular shape in lateral view. This confirms earlier findings of Pergams & Ashley (2001), who found that skulls of introduced black rats (*Rattus rattus*), house mice (*Mus musculus*) and deer mice (*Peromyscus maniculatus*) exhibit a trend towards the nose becoming longer and the braincase becoming shallower.

The separation in PC1 (dorsal view, lateral view) of specimens from Remote Oceania and New Zealand from the rest of the geographic distribution is in accordance with body mass distributions, where specimens from the former regions are on average larger (Motokawa, Lin & Lu, 2004; Van der Geer, Lomolino & Lyras, 2018; Van der Geer, 2018). Also, the proportional scaling of the foramen magnum with skull size is according to expectations of the existence of a correlation between body mass and skull size (Lyras, 2018), because the diameter of the foramen magnum scales with body mass in rodents (Bertrand, Schillaci & Silcox, 2016). Apart from confirming to CREA, another allometric aspect observed in the Polynesian rats is that larger skulls are more tubular in shape than the smaller skulls, which are more balloon-shaped with a rounder and wider braincase relative to those of large skulls. Also Artois et al. (2018) found that smaller individuals have a relatively larger braincase for a mainland sample from the district of Veal Renh in Cambodia, so this appears a pervasive effect, independent of insularity. A similar difference is also observed between the sexes (sexual dimorphism). The findings presented here cannot confirm the practically non-existent sexual dimorphism in Polynesian rats as noted before (Efford, 1976; Yom-Tov, Yom-Tov & Moller, 1999). Males have on average a slightly longer muzzle and narrower skull, a correlate of their slightly larger body mass (3.4%). In addition, in lateral view, males can be separated from the females by their proportionally longer muzzles, independent of their size.

Another allometric effect noted here is that molar size evolution lacks behind during skull size evolution. Polynesian rats with larger skulls have proportionally smaller molars. This effect has been demonstrated previously for insular dwarf elephants during the first stages of body size evolution (Van der Geer et al., 2016). The results presented here indicate that such a delay may also be the case in initial stages of body size increase (gigantism) on islands. Alternatively, the reduced molar length may be linked to a higher degree of carnivory, as shown in a broad study on rodent dietary habits (Samuels, 2009), but further study is needed to confirm this for these populations.

The effect of ecologically relevant competition and predation on skull shape as revealed by the multivariate regression of shape on centroid size using groupings (95% confidence) for the three different views follows body mass distributions, with increased body mass coinciding with decreased competitor and predator pressure, as predicted by ecological models of the ’island rule’. The observed geographic patterning in morphology is associated with average body size differences between the regions, with New Zealand and Remote Oceania at one side (larger specimens), InterOceania, (Island) Papua New Guinea, the Malukus and Micronesia at the centre, and the Mainland, Philippines-Borneo-Sulawesi and the Southern Malay Archipelago at the other side (smaller specimens). This gradual increase in average size from the mainland into the Pacific and especially into Polynesia and New Zealand confirms earlier findings (Yom-Tov, Yom-Tov & Moller, 1999; Atkinson & Towns, 2001; Van der Geer, Lomolino & Lyras, 2018; Van der Geer, 2018). These latter regions are devoid of native mammals, and the Polynesian rats thus had no ecologically significant competition or predation. Hence, the designation of exulans-only for this part of the Pacific: no other murids, or mammals for that matter, are native here. These islands were, and many still are, home to endemic birds and breeding colonies of sea birds. These provided abundant and easy additional resources to the rats, eventually leading to demise of many island bird species (Steadman, 2006) and considerable increase in body mass of the rats.

Interestingly, skulls from the oceanic archipelago Wallacea are morphologically practically indistinguishable from those from the mainland and the continental shelf islands of Sunda and Melanesia. Only skulls from the oceanic islands of Micronesia and Polynesia can be separated from the rest, although a certain overlap was clearly present. This was already noted by Motokawa, Lin & Lu (2004), based on 18 linear morphometric characters from a large sample. On a smaller regional scale (northern Thailand), Claude (2013) noticed shape differences between localities for Polynesian rats, but such differences inevitably disappear here due to a different scale of comparison. Further study is needed to zoom in on the various regions to highlight and analyse the effect of locality. This is beyond the scope of this contribution, which aims at quantifying changes on a larger scale, with the unavoidable loss of such detail. Nevertheless, a clear ecological similarity exists between these regions, apart from Micronesia and Polynesia: namely, they all harbour a multitude of native murids. The Polynesian rat, whether introduced or native, has to compete here with many species, and this, presumably, has an effect on size and shape. Elsewhere, Polynesian rats experienced ecological release from such competition and had the opportunity to explore different ecological niches and increase their resource breadth.

Rats from Micronesia and Polynesia were introduced last, and have thus spent the shortest time on their respective islands. This confirms the notion of rapid evolution in alien rodents on islands (e.g., Berry, 1964; Pergams & Ashley, 2001; Jones, Chown & Gaston, 2003; Cucchi et al., 2014; Ledevin et al., 2016; Renaud et al., 2018), where often one or two centuries are already sufficient to evolve considerably different skull shapes. Also, it indicates that this short span evolution mainly concerns body size evolution without restructuring. Skulls from these latter islands also differ in the range of variation in dorsal PC2 shape space, and this coincides with the distribution of the mtDNA haplotypes (West et al., 2017): the variation is lowest in specimens with haplotype 3 relative to those with haplotypes 1 and 3. This shows that the rats from Remote Oceania and New Zealand are more uniform in shape with narrower temporal regions, resulting in a clearly tubular shape. This loss of shape variation may be a result of the coinciding size increase, or with a loss of genetic variation.

## Conclusion

Introduced Polynesian rats evolve skull shapes that conform to the general mammalian interspecific pattern of cranial evolutionary allometry (CREA), with proportionally longer snouts in larger specimens. This robust allometric relationship is captured best in the first principal component in all three skull views (dorsal, lateral, ventral). Another allometric aspect observed in the Polynesian rats is that larger skulls are more tubular in shape than the smaller skulls, which are more balloon-shaped with a rounder and wider braincase relative to those of large skulls. This difference is also observed between the sexes (sexual dimorphism), due to the slightly larger average male size. The molar teeth are relatively smaller in larger skulls, confirming earlier observations that skull size initially evolves faster than teeth size. Skull size evolution on islands up to twice the size observed in mainland populations in Polynesian rats is mainly influenced by the number of ecologically relevant competitors and predators (native biodiversity) and explains the more tubular skulls with long snouts in Polynesia, where no native mammals occur naturally.

##  Supplemental Information

10.7717/peerj.9076/supp-1 Data S1 Specimen numbers and variables of Polynesian rats used in this study This file contains specimen numbers of skulls of Rattus exulans examined at the American Museum of Natural History (New York; AMNH), the Smithsonian Institute, Natural History Museum (Washington; USNM) and Te Papa Tongarewa Museum of New Zealand (Wellington; NMNZ), as well as geographical and ecological data used as variables in the multivariate analyses.

10.7717/peerj.9076/supp-2 Data S2 Raw data for geometric morphometric assessment of Polynesian rat skull disparity, dorsal aspect Raw two-dimensional coordinates for each landmark (*x*, *y*) for the dorsal skull view for each specimen. Abbreviation code (ID) reflects archipelago (digit one and two), island (digit three and four), museum collection (digit five), gender (last digit), ontogenetic stage (penultimate digit, here always a = adult), and museum registration number (between digit five and the penultimate digit). Example, specimen ID airia99795am is an adult male from the Austral Islands (ai), Raivavae island (ri), curated at the AMNH (a) with number 99795.

10.7717/peerj.9076/supp-3 Data S3 Raw data for geometric morphometric assessment of Polynesian rat skull disparity, lateral aspect Raw two-dimensional coordinates for each landmark (*x*, *y*) are given for the lateral skull view for each specimen. Abbreviation code as in Data S2.

10.7717/peerj.9076/supp-4 Data S4 Raw data for geometric morphometric assessment of Polynesian rat skull disparity, ventral aspect Raw two-dimensional coordinates for each landmark (*x*, *y*) are given for the ventral skull view for each specimen. Abbreviation code as in Data S2.

10.7717/peerj.9076/supp-5 Table S1 Definitions of landmark positions used in this study for geometric morphometric analysis

10.7717/peerj.9076/supp-6 Figure S1 Regional groupings used as independent variable Borneo, the Philippines and Sulawesi (shades of purple) are combined into one region (PhBS), while New Zealand (centre, below, in dark blue) is added. Regional groupings follow Hingston (2015).

10.7717/peerj.9076/supp-7 Figure S2 Shape variation in Canonical Variate Analysis (CVA) (A–D) Dorsal aspect. (E–H) Lateral aspect. (A) CV1 , first axis, regional groupings. (B) CV2, second axi s, regional groupings. (C) CV1, first axis, gender. (D) CV2, second axis, gender. (E) CV1, first axis, regional groupings, simplified. (F) CV2, second axis, regional groupings, simplified. (G) CV1, first axis, gender. (H) CV2, second axis, gender.

10.7717/peerj.9076/supp-8 Figure S3 The effect of native biodiversity on overall skull shape Dorsal aspect, canonical variate analysis . (A) The effect of native competition. Red = 0 competitors, blue = 1 to 4, black = 5 or more competitors. (B) The effect of native predation. Red = 0 predators, blue = 1 or 2, black = 3 or more predators. (C) The overall effect of biodiversity. Red = *exulans* only islands (no native mammals), blue = species poor, black = species rich.

10.7717/peerj.9076/supp-9 Figure S4 The effect of geography and phylogeny on PC1 skull shape Lateral aspect of the skull. (A) The clustering follows the three main regions, with the largest specimens having the lowest PC1 score. Black = Mainland, Philippines-Borneo-Sulawesi, Sunda Shelf. Blue = New Zealand, Remote Oceania. Red = Wallacea, (Island) Papua New Guinea, Near Oceania. (B) The clustering follows that of subspecies with great overlap.

10.7717/peerj.9076/supp-10 Figure S5 Regressions of principal component vectors, ventral view (A) PC1 over latitude, *r*2 = 0.065, Pearson *r* = 0.25. (B) PC2 over latitude, *r*2 = 0.035, Pearson *r* = 0.19. (C) PC2 over latitude, *r*2 = 0.069. Pearson *r* = 0.26. (D) PC3 is not correlated with centroid size, *p* = 0.76.

## Additional Information and Declarations

### Data Availability

The following information was supplied regarding data availability: The specimen data and associated ecological and geographic data are available in Data S1. The raw coordinates of the landmarks for the three skull views (dorsal, lateral, ventral) are available in Data S2–S4 respectively.

## References

[ref-1] Artois J, Blasdell K, Duong V, Buchy P, Hul V, Morand S, Claude J (2018). Effects of mammarenavirus infection (Wẽnzhõu virus) on the morphology of *Rattus exulans*. Infection, Genetics and Evolution.

[ref-2] Atkinson IAE, Towns DR (2001). Advances in New Zealand mammalogy 1990–2000: Pacific rat. Journal of the Royal Society of New Zealand.

[ref-3] Berry RJ (1963). Epigenetic polymorphism in wild populations of *Mus musculus*. Genetics Research.

[ref-4] Berry RJ (1964). The evolution of an island population of the house mouse. Evolution.

[ref-5] Berry RJ (1969). History in the evolution of *Apodemus sylvaticus* (Mammalia) at one edge of its range. Journal of Zoology, London.

[ref-6] Berry RJ, Peters J (1975). Macquarie island house mice: a genetical isolate on a sub-Antarctic island. Journal of Zoology, London.

[ref-7] Bertrand OC, Schillaci MA, Silcox MT (2016). Cranial dimensions as estimators of body mass and locomotor habits in extant and fossil rodents. Journal of Vertebrate Paleontology.

[ref-8] Boyle A, Swann G, Willing T, Gale T, Collins L (2004). Adele Island bird survey report. Unpublished report.

[ref-9] Cardini A (2014). Missing the third dimension in geometric morphometrics: how to assess if 2D images really are a good proxy for 3D structures?. Hystrix.

[ref-10] Cardini A (2019). Craniofacial allometry is a rule in evolutionary radiations of placentals. Evolutionary Biology.

[ref-11] Cardini A, Polly PD (2013). Larger mammals have longer faces because of size-related constraints on skull form. Nature Communications.

[ref-12] Claude J (2013). Log-shape ratios, procrustes superimposition, elliptic fourier analysis: three worked examples in R. Hystrix, Italian Journal of Mammalogy.

[ref-13] Corbet GB, Hill JE (1992). The mammals of the Indomalayan region: a systematic review.

[ref-14] Corti M, Rohlf FJ (2001). Chromosomal speciation and phenotypic evolution in the house mouse. Biological Journal of the Linnean Society.

[ref-15] Cucchi T, Barnett R, Martínková N, Renaud S, Renvoisé E, Evin A, Sheridan A, Mainland I, Wickham-Jones C, Tougard C, Quéré JP, Pascal M, Pascal M, Heckel G, O’Higgins P, Searle JB, Dobney KM (2014). The changing pace of insular life: 5,000 years of microevolution in the Orkney vole (*Microtus arvalis orcadensis*). Evolution.

[ref-16] Cucchi T, Kovács ZE, Berthon R, Orth A, Bonhomme F, Evin A, Siahsarvie R, Darvish J, Bakhshaliyev V, Marro C (2013). On the trail of Neolithic mice and men towards Transcaucasia: zooarchaeological clues from Nakhchivan (Azerbaijan). Biological Journal of the Linnean Society.

[ref-17] Cucchi T, Orth A, Auffray J-C, Renaud S, Fabre L, Catalan J, Hadjisterkotis E, Bonhomme F, Vigne J-D (2006). A new endemic species of the subgenus *Mus* (Rodentia, Mammalia) on the Island of Cyprus. Zootaxa.

[ref-18] Danielsen E, Balete DS, Christensen TD, Hoegaard M, Jakobsen OF, Jensen A, Luns T, Poulsen MK (1994). Conservation of biological diversity in the Sierra Madre Mountains of Isabela and Southern Cagayan Province, the Philippines.

[ref-19] Dryden IL, Mardia KM (2008). Statistical shape analysis.

[ref-20] Dwyer P (1978). A study of *Rattus exulans* in the New Guinea highlands. Australian Wildlife Research.

[ref-21] Efford M (1976). *Rattus exulans* in Polynesia—a case of morphometric divergence.

[ref-22] Gould SJ (1966). Allometry and size in ontogeny and phylogeny. Biological Reviews.

[ref-23] Gould SJ (2002). The structure of evolutionary theory.

[ref-24] Gould SJ, Raup DM, Sepkoski JJ, Schopf TJM, Simberloff DS (1977). The shape of evolution: a comparison of real and random clades. Paleobiology.

[ref-25] Hammer Ø, Harper DAT, Ryan PD (2001). PAST: paleontological statistics software package for education and data analysis. Palaeontologia Electronica.

[ref-26] Hingston M (2015). Phylogeography of the commensal Rattus exulans with implications for its use as bioproxy for human migrations. PhD Thesis.

[ref-27] Hulme-Beaman A, Claude J, Chaval Y, Evin A, Morand S, Vigne J-D, Dobney K, Cucchi T (2019). Dental shape variation and phylogenetic signal in the Rattini tribe species of Mainland Southeast Asia. Journal of Mammalian Evolution.

[ref-28] Hulme-Beaman A, Cucchi T, Evin A, Searle JB, Dobney K (2018). Exploring *Rattus praetor* (Rodentia, Muridae) as a possible species complex using geometric morphometrics on dental morphology. Mammalian Biology.

[ref-29] Jones AG, Chown SL, Gaston KJ (2003). Introduced house mice as a conservation concern on Gough Island. Biodiversity and Conservation.

[ref-30] Karnoukhova NG (1971). Age determination of brown and black rats. The Soviet Journal of Ecology.

[ref-31] Kawakami M, Yamamura K-I (2008). Cranial bone morphometric study among mouse strains. BMC Evolutionary Biology.

[ref-32] Kendall DG (1984). Shape manifolds, procrustean metrics, and complex projective spaces. Bulletin of the London Mathematical Society.

[ref-33] King C (2006). The handbook of New Zealand mammals.

[ref-34] Klingenberg CP (2011). MorphoJ: an integrated software package for geometric morphometrics. Molecular Ecology Resources.

[ref-35] Klingenberg CP, Barluenga M, Meyer A (2002). Shape analysis of symmetric structures: quantifying variation among individuals and asymmetry. Evolution.

[ref-36] Ledevin R, Chevret P, Ganem G, Britton-Davidian J, Hardouin EA, chapuis J-L, Pisanu B, Da Luz Mathias M, Schlager S, Auffray J-C, Renaud S (2016). Phylogeny and adaptation shape the teeth of insular mice. Proceedings of the Royal Society B.

[ref-37] Li J, Huang J-P, Sukumaran J, Knowles LL (2018). Microevolutionary processes impact macroevolutionary patterns. BMC Evolutionary Biology.

[ref-38] Lomolino MV (1985). Body size of mammals on islands: the island rule re-examined. American Naturalist.

[ref-39] Lomolino MV (2005). Body size evolution in insular vertebrates: generality of the island rule. Journal of Biogeography.

[ref-40] Lomolino MV, Van der Geer AAE, Lyras GA, Palombo MR, Sax D, Rozzi R (2013). Of mice and mammoths: generality and antiquity of the island rule. Journal of Biogeography.

[ref-41] Lomolino MV, Riddle BR, Whittaker RJ (2017). Biogeography. Biological diversity across space and time.

[ref-42] Lomolino MV, Sax DF, Palombo MR, Van der Geer AA (2012). Of mice and mammoths: evaluations of causal explanations for body size evolution in insular mammals. Journal of Biogeography.

[ref-43] Lyras GA (2018). Brain changes during phyletic dwarfing in elephants and hippos. Brain Behaviour and Evoloution.

[ref-44] Maestri R, Patterson BD, Fornel R, Monteiro LR, De Freitas TRO (2016). Diet, bite force and skull morphology in the generalist rodent morphotype. Journal of Evolutionary Biology.

[ref-45] Mardia KV, Bookstein FL, Moreton IJ (2000). Statistical assessment of bilateral symmetry of shapes. Biometrika.

[ref-46] Matisoo-Smith E, Robins JH, Green RC (2004). Origins and dispersals of Pacific peoples: evidence from mtDNA phylogenies of the Pacific rat. Proceedings of the National Academy of Sciences of the United States of America.

[ref-47] Millien V (2006). Morphological evolution is accelerated among island mammals. PLOS Biology.

[ref-48] Molur S, Srinivasulu C, Srinivasulu B, Walker S, Nameer PO, Ravikumar L (2005). Status of non-volant small mammals: conservation assessment and management plan (C.A.M.P) workshop report.

[ref-49] Motokawa M, Lin L-K, Lu K-H (2004). Geographic variation in cranial features of the Polynesian rat *Rattus exulans* (Peale, 1848) (Mammalia: Rodentia: Muridae). The Raffles Bulletin of Zoology.

[ref-50] Motokawa M, Lu K-H, Harada M, Lin L-K (2001). New records of the Polynesian rat *Rattus exulans* (Mammalia: Rodentia) from Taiwan and the Ryukyus. Zoological Studies.

[ref-51] Musser GG (1981). The giant rat of Flores and its relatives east of Borneo and Bali. Bulletin of the American Museum of Natural History.

[ref-52] Musser GG, Carleton MD, Wilson DE, Reeder DA (2005). Superfamily Muroidea. Mammal species of the world: a geographic and taxonomic reference.

[ref-53] Oliver JC (2013). Microevolutionary processes generate phylogenomic discordance at ancient divergences. Evolution.

[ref-54] Patton JL, Yang SY, Myers P (1975). Genetic and morphological divergence among introduced rat populations (*Rattus rattus*) of the Galapagos archipelago, Equador. Systematic Zoology.

[ref-55] Pergams ORW, Ashley MV (2001). Microevolution in island rodents. Genetica.

[ref-56] Phylopic (2020). http://phylopic.org/image/570c7d9e-e6d1-46f5-b165-988981bfc5f6/.

[ref-57] Pietrusewsky M, Katzenberg MA, Saunders RA (2008). Metric analysis of skeletal remains: methods and applications. Biological anthropology of the human skeleton.

[ref-58] Rabor DS (1977). Philippine birds and mammals.

[ref-59] Radinsky LB (1985). Approaches in evolutionary morphology: a search for patterns. Annual Review of Ecology and Systematics.

[ref-60] Renaud S, Ledevin R, Souquet L, Rodrigues HG, Ginot S, Agret S, Claude J, Herrel A, Hautier L (2018). Evolving teeth within a stable masticatory apparatus in Orkney mice. Evolutionary Biology.

[ref-61] Roach HI, Mehta G, Oreffo RO, Clarke NM, Cooper C (2003). Temporal analysis of rat growth plates, cessation of growth with age despite presence of a physis. Journal of Histochemistry & Cytochemistry.

[ref-62] Roberts M (1991). Origin, dispersal routes, and geographic distribution of Rattus exulans, with special reference to New Zealand. Pacific Science.

[ref-63] Rohlf FJ (2006). http://life.bio.sunysb.edu/morph/index.html.

[ref-64] Rohlf FJ, Marcus LF (1993). A revolution in morphometrics. Trends in Ecology and Evolution.

[ref-65] Samuels JX (2009). Cranial morphology and dietary habits of rodents. Zoological Journal of the Linnean Society.

[ref-66] Schluter D (1996). Adaptive radiation along genetic lines of least resistance. Evolution.

[ref-67] Schwarz E (1960). Classification, origin and distribution of commensal rats. Bulletin of the World Health Organization.

[ref-68] Schwarz E, Schwarz HK (1967). A monograph of the *Rattus rattus* group. Annals Escuela Nacional Ciencias Biologicas.

[ref-69] Sepkoski D (2012). Rereading the fossil record: the growth of paleobiology as an evolutionary discipline.

[ref-70] Shapiro SS, Wilk MB (1965). An analysis of variance test for normality (complete samples). Biometrica.

[ref-71] Singh N, Albert FW, Plyusnina I, Trut L, Pääbo S, Harvati K (2017). Facial shape differences between rats selected for tame and aggressive behaviors. PLOS ONE.

[ref-72] Smith HF (2011). The role of genetic drift in shaping modern human cranial evolution: a test using microevolutionary modeling. International Journal of Evolutionary Biology.

[ref-73] Steadman DW (2006). Extinction and biogeography of tropical pacific birds.

[ref-74] Tamagnini D, Meloro C, Cardini A (2017). Anyone with a long-face? Craniofacial Evolutionary Allometry (CREA) in a family of short-faced mammals, the Felidae. Evolutionary Biology.

[ref-75] Tate GHH (1935). Rodents of the genera *Rattus* and *Mus* from the Pacific Islands, collected by the Whitney South Sea expedition, with a discussion of the origin and races of the Pacific Island rat. Bulletin of the American Museum of Natural History.

[ref-76] Thomson V, Aplin KP, Cooper A, Hisheh S, Suzuki H, Maryanto I, Yap G, Donnellan SC (2014). Molecular Genetic Evidence for the Place of Origin of the Pacific Rat, *Rattus exulans*. PLOS ONE.

[ref-77] Valenzuela S, Poitevin F, Cornette R, Bournery A, Nada J, Vigne J-D (2009). Evolving ecosystems: ecological data from an Iron Age small mammal accumulation at Alorda Park (Catalonia, Spain). Journal of Archaeological Science.

[ref-78] Van der Geer AAE (2018). Changing invaders: trends of gigantism in insular introduced rats. Environmental Conservation.

[ref-79] Van der Geer AAE, Van den Bergh G, Lyras GA, Prasetyo UW, Due RA, Setiyabudi E, Drinia H (2016). The effect of area and isolation on insular dwarf proboscideans. Journal of Biogeography.

[ref-80] Van der Geer AAE, Lomolino MV, Lyras GA (2017). Islands before man: the species–area relationship during the late Pleistocene. Journal of Biogeography.

[ref-81] Van der Geer AAE, Lomolino MV, Lyras GA (2018). On being the right size—do aliens follow the rules. Journal of Biogeography.

[ref-82] Van Valen L (1962). A study of fluctuating asymmetry. Evolution.

[ref-83] West K, Collins C, Kardailsky O, Kahn J, Hunt TL, Burley DV, Matisoo-Smith E (2017). The Pacific rat race to Easter Island: tracking the prehistoric dispersal of *Rattus exulans* using ancient mitochondrial genomes. Frontiers in Ecology and Evolution.

[ref-84] Whittaker RJ, Fernandez-Palacios JM (2007). Island biogeography. Ecology, evolution, and conservation.

[ref-85] Williams JM (1973). The ecology of *Rattus exulans* (Peale) reviewed. Pacific Science.

[ref-86] Wilmshurst JM, Anderson AJ, Higham TFG, Worthy TH (2008). Dating the late prehistoric dispersal of Polynesians to New Zealand using the commensal Pacific rat. Proceedings of the National Academy of Sciences of the United States of America.

[ref-87] Wilmshurst JM, Hunt TL, Lipo CP, Anderson AJ (2011). High-precision radiocarbon dating shows recent and rapid initial human colonization of East Polynesia. Proceedings of the National Academy of Sciences of the United States of America.

[ref-88] Yom-Tov Y, Yom-Tov S, Moller H (1999). Competition, coexistence and adaptation among rodent invaders of Pacific and New Zealand islands. Journal of Biogeography.

[ref-89] Zelditch ML, Swiderski DL, Sheets HD (2012). Geometric morphometrics for biologists: a primer.

