# Supplementary material for: Size matters: micro-evolution in Polynesian rats highlights body size changes as initial stage in evolution
Source: PeerJ. 2020 Apr 28;8:e9076. doi: 10.7717/peerj.9076 (PMC7194086; doi:10.7717/peerj.9076)
Supplement: Table S1 [file peerj-08-9076-s005.docx]

Table S1. Definitions of landmark positions used in this study for geometric morphometric analysis.

| Dorsal aspect | 1 | Anteriormost tip of the median line |
| --- | --- | --- |
|  | 2, 3 | Anteriormost contact between the nasal and premaxillar bone |
|  | 4, 6 | Orbital end of the premaxillary-frontal suture |
|  | 5 | Midpoint intersection of the nasal bones (nasion) |
|  | 7, 9 | Lateral end of the temporal-frontal suture |
|  | 8 | Midpoint intersection of the coronal and sagittal sutures (bregma) |
|  | 10, 12 | Anterolateral tip of the interparietal bones |
|  | 11 | Midpoint intersection of the temporal and occipital bones (lambda) |
|  | 13 | Midpoint intersection of the interparietal-occipital sutures |
|  | 14, 15 | Occipital condyle |
| Lateral aspect | 1 | Anterior tip of the nasal bones |
|  | 2 | Anteriormost point of the premaxillary-nasal suture |
|  | 3 | Inferiormost point of the incisive alveole |
|  | 4 | Posteriormost point of the incisive alveole |
|  | 5 | Dorsalmost point above M2-M3 contact |
|  | 6 | Lateral end of the frontal-parietal suture |
|  | 7 | Dorsalmost point of frontal-parietal suture |
|  | 8 | Dorsalmost point of parietal-interparietal suture |
|  | 9 | Posteriormost end of interparietal bones |
|  | 10 | Ventralmost end of interparietal bones |
|  | 11 | Contact point between the occipital condyle and the occipital bone |
|  | 12 | Tip of styloid process of the temporal bone |
|  | 13 | Posteriormost contact between zygomatic bone and the zygomatic process of the temporal bone |
|  | 14 | Posteriormost point of the molar row |
|  | 15 | Anteriormost point of the molar row |
| Ventral aspect | 1 | Anteriormost contact of the nasal bones |
|  | 2 | Midpoint between the incisors, proximal end |
|  | 3, 4 | Lateralmost point of the incisor alveole |
|  | 5 | Midpoint of anteriormost end of incisive foramen |
|  | 6 | Midpoint of posteriormost end of incisive foramen |
|  | 7, 29 | Anteriormost tip of the zygomatic arch |
|  | 8, 27 | Anteriormost point of the molar row |
|  | 9, 26 | Lateral contact between the first and second molar |
|  | 10, 25 | Posteriormost point of the molar row |
|  | 11, 28 | Lateralmost point of the zygomatic arch |
|  | 12, 23 | Anteriormost point of the auditory bulla |
|  | 13, 22 | Anteriormost external border of the ectotympanic bone |
|  | 14, 21 | Posteriormost point of the auditory bulla |
|  | 15, 17 | Medioposterior point of the occipital condyle |
|  | 16 | Posteriormost point of the foramen magnum on the midline |
|  | 18 | Anteriormost point of the foramen magnum on the midline |
|  | 19, 20 | Contact point of basisphenoid-basioccipital suture and auditory bulla |
|  | 24 | Posteriormost extent of the palate at the midline |
