# Supplementary figures and images for: Size matters: micro-evolution in Polynesian rats highlights body size changes as initial stage in evolution
Source: PeerJ. 2020 Apr 28;8:e9076. doi: 10.7717/peerj.9076 (PMC7194086; doi:10.7717/peerj.9076)

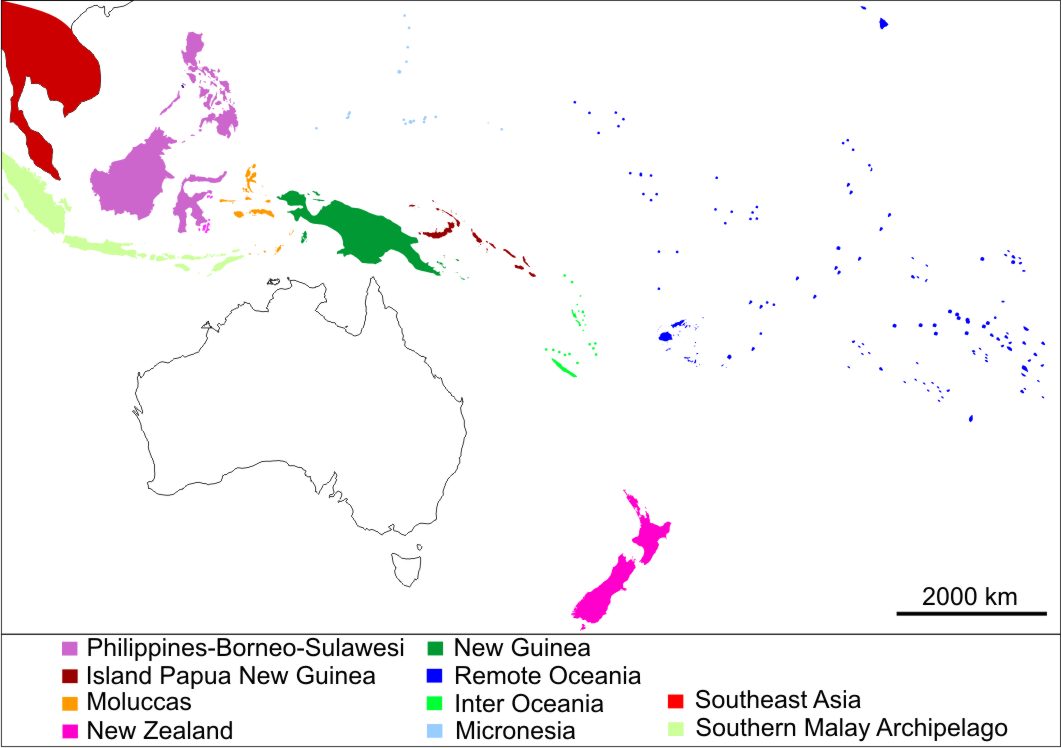

Supplement: Figure S1 — Borneo, the Philippines and Sulawesi (shades of purple) are combined into one region (PhBS), while New Zealand (centre, below, in dark blue) is added. Regional groupings follow Hingston (2015). [file peerj-08-9076-s006.jpg]

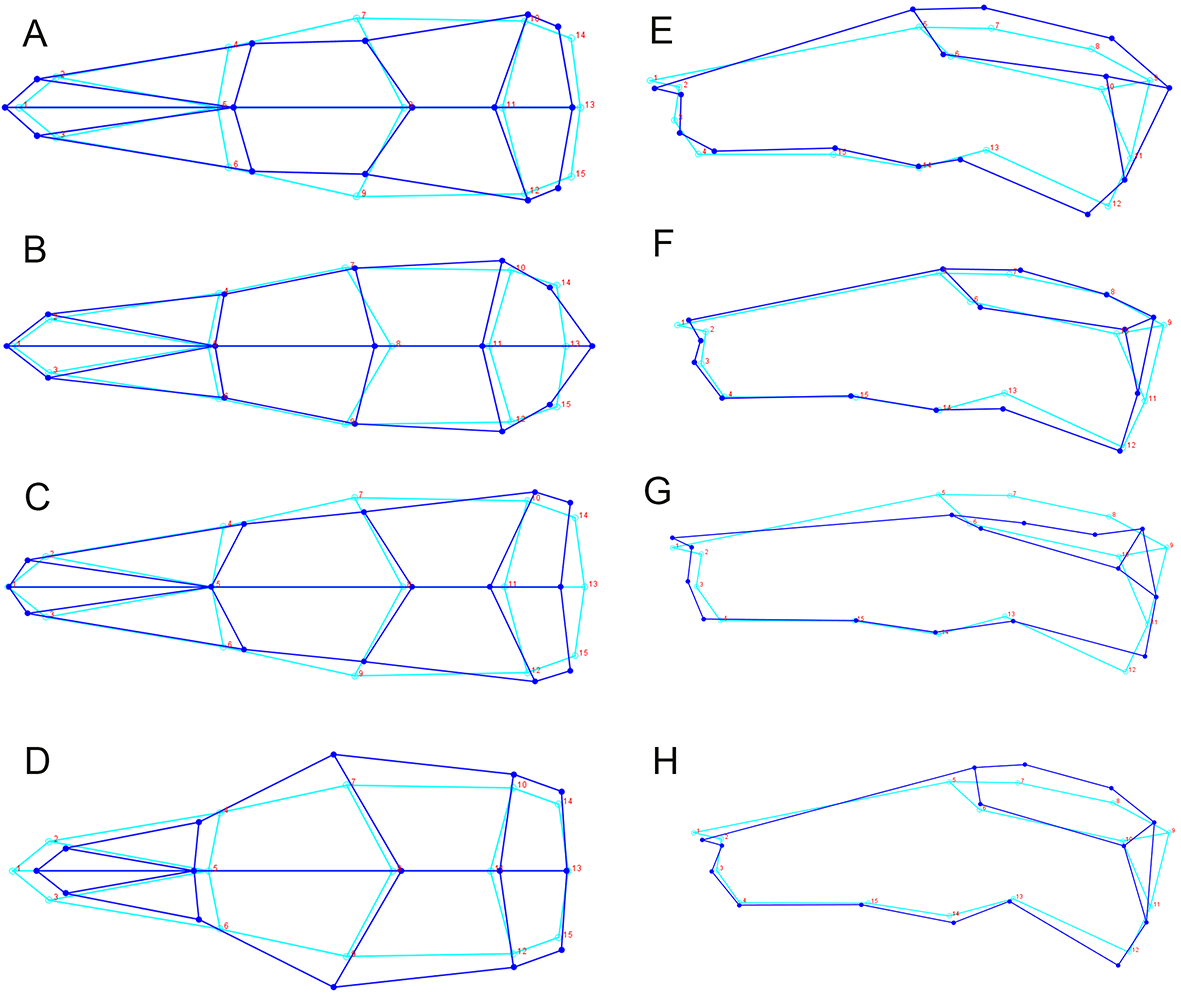

Supplement: Figure S2 — (A–D) Dorsal aspect. (E–H) Lateral aspect. (A) CV1 , first axis, regional groupings. (B) CV2, second axi s, regional groupings. (C) CV1, first axis, gender. (D) CV2, second axis, gender. (E) CV1, first axis, regional groupings, simplified. (F) CV2, second axis, regional groupings, simplified. (G) CV1, first axis, gender. (H) CV2, second axis, gender. [file peerj-08-9076-s007.png]

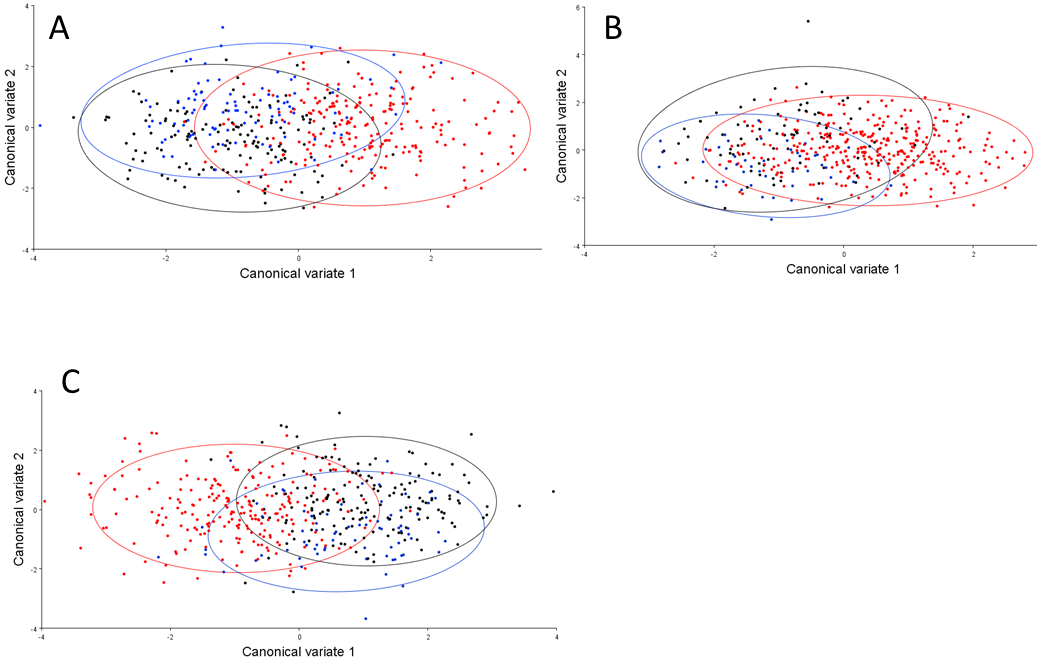

Supplement: Figure S3 — Dorsal aspect, canonical variate analysis . (A) The effect of native competition. Red = 0 competitors, blue = 1 to 4, black = 5 or more competitors. (B) The effect of native predation. Red = 0 predators, blue = 1 or 2, black = 3 or more predators. (C) The overall effect of biodiversity. Red = exulans only islands (no native mammals), blue = species poor, black = species rich. [file peerj-08-9076-s008.png]

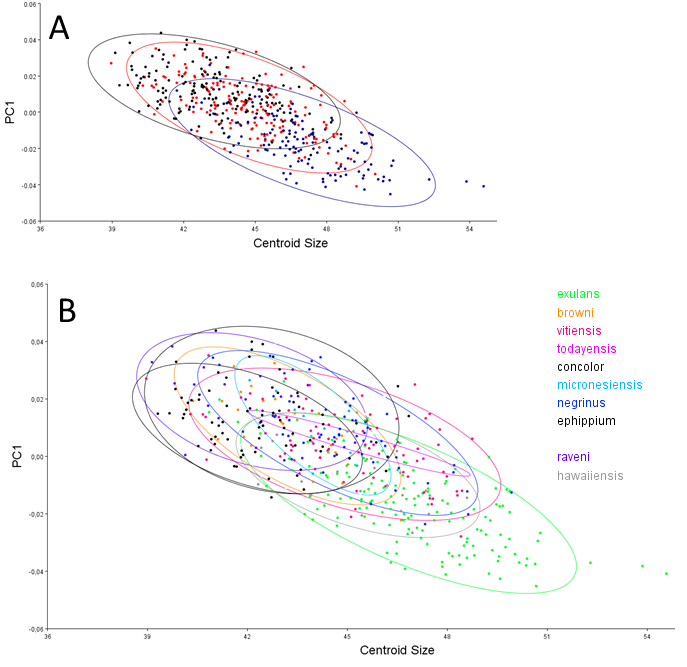

Supplement: Figure S4 — Lateral aspect of the skull. (A) The clustering follows the three main regions, with the largest specimens having the lowest PC1 score. Black = Mainland, Philippines-Borneo-Sulawesi, Sunda Shelf. Blue = New Zealand, Remote Oceania. Red = Wallacea, (Island) Papua New Guinea, Near Oceania. (B) The clustering follows that of subspecies with great overlap. [file peerj-08-9076-s009.png]

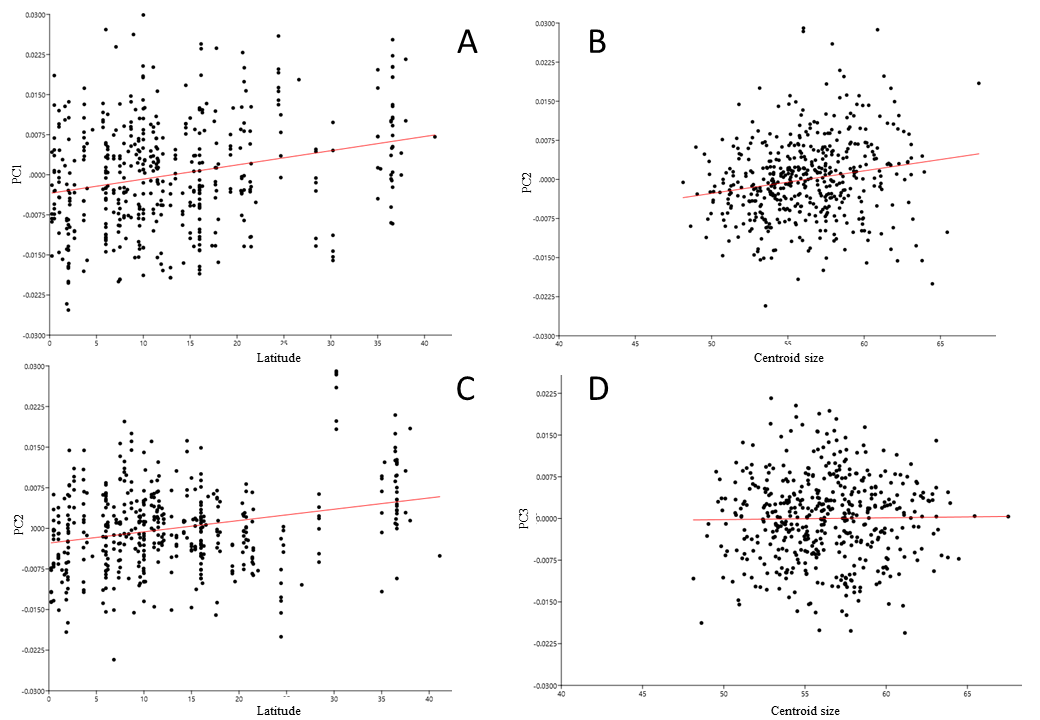

Supplement: Figure S5 — (A) PC1 over latitude, r2 = 0.065, Pearson r = 0.25. (B) PC2 over latitude, r2 = 0.035, Pearson r = 0.19. (C) PC2 over latitude, r2 = 0.069. Pearson r = 0.26. (D) PC3 is not correlated with centroid size, p = 0.76. [file peerj-08-9076-s010.png]
